# Prevalence and associated factors of non-consensual sexual acts among adolescents in the Democratic Republic of Congo

**DOI:** 10.3389/frph.2024.1437225

**Published:** 2024-12-11

**Authors:** Miangotar Yode, Felly Ekofo, Blaise Mudekereza Mihigo

**Affiliations:** ^1^ Center for Research and Study of African Populations and Societies, University of N'Djamena, N'Djamena, Chad; ^2^ National Program of AIDS Control, Kinshasa, Democratic Republic of Congo; ^3^ CORDAID, Kinshasa, Democratic Republic of Congo

**Keywords:** sexuality, non-consensual sexual, forced sexual, adolescents, Democratic Republic of Congo

## Abstract

**Introduction:**

During adolescence, due to lack of experience, individuals may engage in or tolerate certain non-consensual acts under coercion. There are significant associations between forced sexual intercourse and a range of negative effects on reproductive health, as well as psychological and emotional health. Studies on non-consensual sexual acts among adolescents are rare in the Democratic Republic of Congo (DRC). This study is one of the first to focus on urban adolescents, aiming to assess the prevalence of non-consensual sexual acts and to identify associated factors.

**Methods:**

Data for this study were obtained from the baseline survey of the project “Reducing the Vulnerability of Adolescents and Young Girls to Violence and HIV/AIDS Infection,” conducted in 2018 in the provinces of Kinshasa and Kasaï Oriental among adolescents aged 10-24 years. A total of 2,123 adolescents were surveyed (46.8% in Kinshasa and 53.2% in Kasaï Oriental). A structured survey questionnaire was developed in French to collect data. This questionnaire was pre-tested and corrected before use. Non-consensual sexual acts were assessed using three variables: (i) Have you ever experienced non-consensual touching, (ii) Have you ever experienced an attempted forced sexual intercourse, and (iii) Have you ever been physically forced, injured, or threatened to have sexual intercourse. Bivariate and multivariate analyses were conducted on these three variables separately.

**Results:**

Among all adolescents surveyed, 11.5% reported having experienced non-consensual touching, 15.3% reported having experienced an attempted forced sexual intercourse, and 5.8% reported having been physically forced, injured, or threatened to have sexual intercourse. Among the 575 sexually active adolescents, these proportions were 43.4%, 57.4% and 22.0%, respectively. Prevalences were higher among girls and in the province of Kinshasa. Factors associated with non-consensual sexual acts included gender, cohabitation with biological parents (father and mother), age at first sexual intercourse, communication about sexuality or intimate subjects with a family member, and adolescents' perceptions of the role and place of partners in intimate relationships. A quarter (25.5%) of adolescents who were victims of forced sexual intercourse and were aware of an appropriate institution or person sought help from a professional for the violence they experienced.

**Discussion:**

The results revealed a high prevalence of non-consensual sexual acts, the significance attributed by adolescents to traditional beliefs regarding the dominant role of men in intimate relationships, and the existence of barriers preventing victims of sexual violence from seeking appropriate care. These findings advocate for providing healthcare services tailored to the needs of adolescents and adapted to sociocultural contexts.

## Introduction

Non-consensual sexual acts refer to sexual acts experienced under coercion, classified under the category of sexual violence. According to Bagwell-Gray et al. (1), sexual violence can be categorized into four groups: (1) acts of sexual coercion, (2) sexual assaults, (3) abusive sexual behaviors, and (4) forced sexual activities. During adolescence, due to lack of experience, individuals may engage in or tolerate certain non-consensual acts under coercion.

Previous research mostly studied sexual coercion using victimization perspective. The focus is on prevalence, associated factors, and impacts on the individual, family, and society. Verissimo et al. (2) found a prevalence of sexual victimization of 36.4% among adolescents aged 12–19 in a public school in Recife, Brazil. A meta-analysis of 16 reviews reported a prevalence of sexual violence ranging from 0.1% to 64.6% in adolescent and youth romantic relationships (3). Another meta-analysis highlighted that one in seven adolescent girls, aged 13–18, experienced at least one form of sexual violence perpetrated by an intimate partner (4). The authors found an overall prevalence of sexual violence of 9%. Girls reported lower perpetration rates than boys (3% vs. 10%) and higher victimization rates (14% vs. 8%). Some studies have demonstrated the complexity of assessing the extent of sexual violence as many young people struggle to recognize themselves as victims (5).

In sub-Saharan Africa, studies on non-consensual sexual relations in adolescence have reported different prevalence rates. In Rwanda, a survey conducted among sexually active students in upper secondary education revealed that 15.5% reported having experienced forced sexual intercourse (6). In a university community in Benin City, Nigeria, Gharoro et al. (7) found a prevalence of 18.4% of non-consensual sexual intercourse. A study among sexually experienced youth aged 10–24 in Nyeri, Kenya, recorded rates of coerced sexual relations of 21% for women and 11% for men (8).

A considerable proportion of adolescents are at risk of experiencing non-consensual sexual acts within the context of their first romantic relationships (9, 10). In Rakai, Uganda, 14% of sexually active young women aged 15–19 reported experiencing their first sexual intercourse under coercion (11). Using nationally representative surveys conducted in Malawi among girls aged 12–19 in 2004, Moore et al. (12) found that 38% of girls reported being “not at all willing” during their first sexual experience, while 30% of girls in Ghana, 23% in Uganda, and 15% in Burkina Faso were in similar situations. In Ghana, 25% of women aged 12–24 reported that their first sexual intercourse was forced (13). Coercion experienced at sexual initiation is a major determinant of subsequent sexual behaviors.

In Côte d'Ivoire and Burkina Faso, national surveys have been conducted on violence against children and youth, focusing on the prevalence and types of sexual violence experienced. In Côte d'Ivoire, survey results showed that 19.2% of women aged 18–24 and 11.4% of men experienced sexual violence before the age of 18 (14). Prevalences in Burkina Faso are lower than in Côte d'Ivoire. It was found that 3.1% of children aged 12–17 experienced sexual violence in the past 12 months (5.7% for girls compared to 0.8% for boys) (15). Across the results of both surveys, it appears that girls are more exposed than boys to experiencing various forms of non-consensual sexual acts.

Research has reported significant associations between forced sexual intercourse and a range of negative effects on reproductive health, as well as psychological and emotional health (12, 14, 15). Risks to reproductive health related to sexual coercion include sexually transmitted infections, unwanted pregnancies, and the onset of risky behaviors (other non-consensual sexual experiences, multiple partnerships, and unprotected sexual intercourse).

Some studies have examined factors associated with non-consensual sexual relations among adolescents. Thus, it is found that married or previously married women and those not living with a parent or spouse were at significantly higher risk of sexual coercion (8). In a study in Rwanda, sexual victimization was associated with being female and having (had) a concurrent sexual relationship (6).

In the Democratic Republic of Congo (DRC), sexual violence is cited among the 14 reproductive health problems of adolescents within the framework of the 2008 National Adolescent Health Policy (PNSA). This policy considers sexual violence as one of the causes of mental disorders and aims to combat it by implementing the provisions of articles 167 and 168 of Law 06/018 of July 20, 2006, on sexual violence and providing care for adolescent victims. According to a recent study on sexual and reproductive health services in the DRC (16), a very few health centers (22%) offer services for combating sexual and gender-based violence. Additionally, the PNSA does not incorporate prevention against sexual violence into its objectives.

Studies on non-consensual sexual acts among adolescents are scarce in the DRC. In a report jointly produced by UNFPA and PRB in 2012, a prevalence of sexual violence of 36% for those aged 15%–19% and 31% for those aged 20–24 was noted (12). Thus, there is very little evidence of non-consensual sexual acts experienced by adolescents, both in the general population and across different residential settings. This study is one of the first to focus on non-consensual sexual acts among sexually active adolescents of both sexes residing in urban areas of the DRC. It aims to assess the prevalence of non-consensual sexual acts and to identify factors associated with this phenomenon.

## Materials and methods

### Site and type of study

The Democratic Republic of Congo (DRC) covers an area of 2,345,000 square kilometers and consists of 26 provinces, with a population estimated by the United Nations at 96 million inhabitants in 2018. Adolescents (aged 10–24 years) represented nearly one-third of the population (17).

In 2018, a cross-sectional survey was conducted among adolescents in the provinces of Kinshasa and Kasaï Oriental as part of the project “Reducing the vulnerability of adolescents and young girls to violence and HIV/AIDS infection,” implemented by CORDAID with financial support from the Global Fund. The survey aimed to establish the baseline situation of the project, which seeks to improve adolescents' and young women's access to appropriate health services, enhance their knowledge of sexual and reproductive health as well as human rights, and reduce gender-based violence (GBV) in school and professional environments. The project covered 3 urban health zones in each province. These were Kalamu 1, Makala, and Kitambo in the city of Kinshasa (Kinshasa Province) and Diulu, Kansele, and Nzaba in the city of Mbuji-Mayi (Kasaï Oriental Province). The DRC's healthcare system is structured on 3 levels: the central or national level, the intermediate or provincial level, and the peripheral or operational level. The last level is the Health Zone (ZS), which comprises a General Reference Hospital and health areas (AS). Each health area has a health center (CS).

### Data collection tools

A structured survey questionnaire was developed in French to collect data from adolescents. It covered nine thematic modules: (i) characteristics of adolescents, (ii) community environment, (iii) activities and sexual relationships, (iv) HIV/AIDS and other STIs, (v) forced sexual intercourse, (vi) reproduction, (vii) access to health services, (viii) gender-based violence in school, and (ix) access to information on sexual health and gender-based violence. The module addressing forced sexual intercourse was reserved for adolescents who had experienced their first sexual intercourse. In this module, adolescents were initially asked about their perceptions of the roles and powers of partners of each sex in intimate relationships. Subsequently, questions related to experiences of unwanted touching, attempted forced sexual intercourse, sexual intercourse following threat, violence, or injury were addressed. Victims of forced sex were asked about their use of care services and post-exposure prophylaxis for HIV.

The survey questionnaire was translated into Lingala and Tshiluba, which are the main languages spoken, respectively, in the provinces of Kinshasa and Kasaï Oriental. The French and local language versions were used for field agent training, but only the French version was used for data collection after a pre-test in each province.

### Participants and sampling strategy

The survey covered the 6 urban health zones (3 per province) selected for project implementation. Data were collected from adolescents residing in households within the health areas comprising these health zones. The project targeted individuals aged 10–24 years, who are considered adolescents according to the PNSA document (2008). In the adolescent health policy, adolescence is defined as a period from 10 to 24 years, encompassing early adolescence (10–14 years), adolescence (15–19 years), and late adolescence (20–24 years).

The sample size was determined using the formula below, developed by FANTA (Food and Nutrition Technical Assistance III Project), which is suitable for baseline surveys of projects targeting households. The sheet “Comparative for Proportions” of the Excel file “Population-Based Survey Sampling Calculator” was used to calculate household size (18). The project, whose data were used for this study, aims to reduce the prevalence of risky sexual behaviour in the adolescent population. The formula allowed for the calculation of the minimum number of households to be visited to survey adolescents, considering the average number of adolescents expected per household. The procedure proposed by FANTA makes it easier to determine the size of the sample to be surveyed at the end of the project as part of the final evaluation, with regard to the level of the indicator to be achieved by the project.

$$\begin{array}{rl} n= & \left(D*{\left[\frac{Z_{1-\alpha }\sqrt{2\bar{P}(1-\bar{P})}+Z_{1-\beta }\sqrt{P_{1}(1-P_{1})+P_{2}(1-P_{2})}}{\delta }\right]}^{2}\right)\\ & *\mathrm{ad}j_{1}*\mathrm{ad}j_{2} \end{array}$$

$$\bar{P}=\frac{P_{1}+P_{2}}{2}$$

Where

• n: Minimum sample size.
• D: Effect of the survey sampling design.
• $P_{1}\;$: Level of the indicator (proportion) at the baseline survey.
• $P_{2}$: Expected level of the indicator at the end of the project.
• $Z_{1-\alpha }\;$: Confidence level desired to conclude that an observed change in size (P2—P1) did not occur by chance.
• $Z_{1-\beta }\;$: Confidence level desired to reliably detect a change in size (P2—P1) if such a change did occur.
• δ**:** Minimum size of change to be achieved by the end of the project.
• $\mathrm{ad}j_{1}\;$**:** Adjustment factor for the sample size to account for the level of measurement of the indicator (household or individual). If the indicator is measured at the individual level, the adjustment considers the proportion of the target population and the household size.
• $\mathrm{ad}j_{2}\;$**:** Adjustment factor for the sample size to account for non-responses.

The final sample size of households calculated, based on available data and basic parameters, was 747 households, rounded to 750. It was expected to survey a minimum of 1,500 adolescents in these households. Finally, 2,123 adolescents were surveyed, with 46.8% in the province of Kinshasa and 53.2% in Kasaï Oriental. An excess of the expected target quantities was recorded through a conversion of the planned days for household enumeration into survey time. Initially, the survey plan aimed to allocate 2 days per province for household enumeration to establish a sampling frame. However, once in the field, data on streets and the number of plots per street were used to draw the household sample, bypassing a household enumeration phase.

The sampling of households and adolescents was done randomly. In each province, the number of households to be sampled per health zone was proportional to the demographic weight across the three zones. In each health zone, 3–4 health areas were selected. Within each selected health area, 3–4 streets were randomly chosen, and within the selected street, at least 10 plots, and one household per plot were randomly selected for survey. In the selected household, the head of the household or their representative, as well as all adolescents in the household, were surveyed.

### Data collection, entry, processing, and analysis

The survey process was supervised by a steering committee and a technical committee, chaired by the National Program of AIDS Control.

A team of supervisors, controllers, and investigators (men and women) was recruited and trained in each province for data collection using two paper questionnaires, one addressed to household heads to identify eligible individuals and the second targeting adolescents present in households. Data collection took place in August 2018.

Data entry was conducted in Kinshasa in September 2018 by a team of recruited and trained agents. The data entry template was designed using EPIDATA. The data were exported to SPSS and cleaned using the same software to address inconsistencies not detected during data entry and filters not adhered to in the field. It was also during this phase that responses to open-ended questions (especially for the “Other” categories) were coded. The coding was done after grouping the responses treating the same idea, followed by the determination of a word or group of words best summarizing the idea. This word or group of words was coded and added to the variable's modalities.

Cross-tabulation was used to describe the sociodemographic characteristics of adolescents, assess the prevalence of non-consensual sexual experiences, and describe the use of appropriate services for the care of victims of sexual violence. Non-consensual sexual acts were evaluated using three binary (Yes/No) variables: (i) Ever experienced unwanted touching, (ii) Ever experienced attempted forced sexual intercourse, and (iii) Ever been physically forced, injured, or threatened to have sexual intercourse. Binomial logistic regression was used to model associations between non-consensual sexual acts and sociodemographic characteristics (gender, age group, marital status, education level, cohabitation with biological parents, participation in religious services, internet access, membership in a social group), communication about sexuality or intimate subjects (communication with a family member, communication with a non-family member), and adolescents' perceptions (five statements about trust in their community and power relations in intimate relationships). Due to the small number of sexually active adolescents (575, including 373 in Kinshasa, 64.9%, and 202 in Kasai Oriental, 35.1%), it was not possible to conduct regression models by sex and province.

### Ethical considerations

The survey addressed topics related to adolescents' intimate lives, safety, and social norms. Additionally, it targeted adolescents, who are still immature in certain aspects of life. Given these considerations, measures were taken to avoid risks, protect individuals' rights, and ensure the safety of all study participants. It was through the planning of these measures that the National Ethics Committee for Health (CNES) of the Ministry of Public Health of the Democratic Republic of the Congo gave a favorable opinion to the conduct of the survey (CNES Opinion No. 78/CNES/BN/PMMF/2018 dated 02/08/2018).

## Results

### Sociodemographic characteristics, communication about sexuality or intimate subjects, and adolescents' perceptions

The survey covered adolescents aged 10–24 years, with an average age of 16.3 years. The median age of sexual debut for these adolescents is 15 years (14.7 years for boys and 15.1 years for girls).

More than half (56.2%) of the respondents who had initiated sexual activity were aged 20–24 years, and this was consistent across gender and province (See Table 1). The majority were single (87.5%). Overall, less than a tenth (8.6%) of the respondents had no formal education. However, this proportional distribution was not observed in Kasai Oriental, where 12.9% of the respondents had no education. Thus, most sexually active respondents had attended school, with 84.6% having at least a secondary education level. Three out of ten respondents (30.8%) did not live with either biological parent. The majority (67.0%) participated in religious services (worship/mass/prayer) more than once a week. However, compared to other groups of adolescents, the proportion of boys participating in religious services more than once a week was the lowest (57.6%). Overall, 63.5% of respondents had access (often or rarely) to the internet; male adolescents were proportionally more likely to access the Internet, accounting for 77.1%. Slightly more than three out of ten respondents (31.2%) belonged to a social group (association, group, or club); boys were the most numerous proportionally (39%) to belong to such groups, while girls were the least numerous (26%). Slightly more than half (52.8%) of the respondents communicated about sexuality or intimate subjects with a family member, and the majority (77.4%) did so with a non-family member. Overall, 62.4% and 73.2% of respondents agreed, respectively, with the statements “I think we can trust people in my community” and “I feel safe in my community”. The majority (72.4%) of respondents agreed with the statement “It is the man who decides when to have sexual relations”, 58.7% with the statement “A man needs to have multiple sexual partners, even if he has a regular sexual partner”, and 32.1% with the statement “A woman should tolerate violence from her sexual partner or spouse to avoid losing him”.

**Table 1:** Variables related to sociodemographic characteristics, communication about sexuality or intimate subjects, and perceptions of sexually active adolescents in the provinces of Kinshasa and Kasai oriental (Democratic Republic of the Congo), 2018.

Variables related to sociodemographic characteristics, communication about sexuality or intimate subjects, and adolescents’ perceptions	Overall		Sex				Province			
			Male		Female		Kinshasa		Kasai Oriental	
	*n*	%	*n*	%	*n*	%	*n*	%	*n*	%
Total	575		231	40.2	344	59.8	373	64.9	202	35.1
Sociodemographic characteristics										
Age group										
10–19 years old	252	43.8	95	41.1	157	45.6	158	42.4	94	46.5
20–24 years old	323	56.2	136	58.9	187	54.4	215	57.6	108	53.5
Marital status										
Single	502	87.5	220	95.2	282	82.2	354	95.2	148	73.3
Married/Widowed/Divorced/Separated	72	12.5	11	4.8	61	17.8	18	4.8	54	26.7
Level of education										
No education	49	8.6	19	8.3	30	8.8	23	6.2	26	12.9
Primary	39	6.8	10	4.4	29	8.5	22	6.0	17	8.5
Secondary	372	65.3	153	66.5	219	64.4	240	65.0	132	65.7
Higher education	110	19.3	48	20.9	62	18.2	84	22.8	26	12.9
Cohabitation with biological parents (father and mother)										
Both parents	291	50.6	122	52.8	169	49.1	175	46.9	116	57.4
Father or Mother	107	18.6	42	18.2	65	18.9	86	23.1	21	10.4
None	177	30.8	67	29.0	110	32.0	112	30.0	65	32.2
Participation in religious services (worship/mass/prayer)										
More than once a week	385	67.0	133	57.6	252	73.3	242	64.9	143	70.8
Other	190	33.0	98	42.4	92	26.7	131	35.1	59	29.2
Access to the internet										
Often/Rarely	365	63.5	178	77.1	187	54.4	248	66.5	117	57.9
Never used the internet/Unfamiliar with the internet	210	36.5	53	22.9	157	45.6	125	33.5	85	42.1
Membership in a social group (association, club)										
Yes	179	31.2	90	39.0	89	26.0	118	31.6	61	30.4
No	395	68.8	141	61.0	254	74.1	255	68.4	140	69.7
Communication about sexuality or intimate subjects										
Communication about sexuality or intimate subjects with a family member										
Yes	298	52.8	111	48.9	187	55.5	178	48.8	120	60.3
No	266	47.2	116	51.1	150	44.5	187	51.2	79	39.7
Communication about sexuality or intimate subjects with someone outside the family										
Yes	427	77.4	189	85.1	238	72.1	277	78.0	150	76.1
No	125	22.6	33	14.9	92	27.9	78	22.0	47	23.9
Adolescents' perceptions										
I believe we can trust people in my community										
Agree	359	62.4	162	70.1	197	57.3	196	52.6	163	80.7
Disagree	216	37.6	69	29.9	147	42.7	177	47.5	39	19.3
I feel safe in my community										
Agree	420	73.2	185	80.1	235	68.5	258	69.4	162	80.2
Disagree	154	26.8	46	19.9	108	31.5	114	30.7	40	19.8
It's the man who decides when to have sexual relations										
Agree	404	72.4	172	77.5	232	69.1	230	63.9	174	87.9
Disagree	154	27.6	50	22.5	104	31.0	130	36.1	24	12.1
A man needs to have multiple sexual partners, even if he has a regular sexual partner										
Agree	310	58.7	125	59.2	185	58.4	181	53.4	129	68.3
Disagree	218	41.3	86	40.8	132	41.6	158	46.6	60	31.8
A woman should tolerate violence from her sexual partner or spouse to avoid losing him										
Agree	164	32.1	78	38.4	86	27.9	73	22.2	91	50.0
Disagree	347	67.9	125	61.6	222	72.1	256	77.8	91	50.0

### Prevalence of non-consensual sexual acts

Table 2 presents the prevalence of experiences of non-consensual sexual acts among adolescents, for all surveyed adolescents and for those who are sexually active.

**Table 2:** Prevalence of non-consensual sexual acts among adolescents in the provinces of Kinshasa and Kasai oriental (Democratic Republic of the Congo), 2018.

	Among all adolescents			Among adolescents already sexually active		
	Has experienced non-consensual touching	Has experienced an attempted forced sexual intercourse	Has experienced physical force, injury, or threats for sexual intercourse	Has experienced non-consensual sexual touching	Has experienced an attempted forced sexual intercourse	Has experienced physical force, injury, or threats for sexual intercourse
Sex						
Male	10.6	13.9	3.6	39.9	51.5	13.7
Female	12.2	16.3	7.3	45.8	61.5	27.5
Province						
Kinshasa	15.5	21.8	6.8	42.2	59.1	18.5
Kasai Oriental	8.1	9.7	5.0	45.7	54.5	28.3
Overall	11.5	15.3	5.8	43.4	57.4	22.0

Considering all adolescents who participated in the survey, 11.5% reported having experienced unwanted touching, 15.3% reported having experienced attempted forced sexual intercourse, and 5.8% reported having been physically forced, injured, or threatened to have sexual intercourse. These prevalences were higher among girls compared to boys and higher in Kinshasa province than in Kasai Oriental.

Among sexually active adolescents, the levels of prevalence of non-consensual sexual acts were almost quadrupled. In this group, 43.4% reported having experienced unwanted touching, 57.4% reported having experienced attempted forced sexual intercourse, and 22.0% reported having been physically forced, injured, or threatened to have sexual intercourse. As in the overall group of adolescents, prevalences were higher among girls and in Kinshasa province. The only exception was observed for the proportion of adolescents who had been physically forced, injured, or threatened to have sexual intercourse, which was higher in Kasai Oriental (28.3%) than in Kinshasa province (18.5%).

### Characteristics of adolescents involved in non-consensual sexual acts

Table 3 presents the intersection of indicators of non-consensual sexual acts with variables related to sociodemographic characteristics, communication about sexuality or intimate subjects, and adolescents' perceptions. Only associations significant at the 10% threshold are interpreted.

**Table 3:** Experience of non-consensual sexual acts according to variables related to socio-demographic characteristics, communication about sexuality or intimate subjects, and perceptions of adolescents in the provinces of Kinshasa and Kasai oriental (Democratic Republic of the Congo), 2018.

Variables related to sociodemographic characteristics, communication about sexuality or intimate subjects, and adolescents’ perceptions	Has experienced non-consensual touching			Has experienced an attempted forced sexual intercourse			Has experienced physical force, injury, or threats for sexual intercourse		
	Yes		Prob. Chi2	Yes		Prob. Chi2	Yes		Prob. Chi2
	*n*	%		*n*	%		*n*	%	
Total	245			324			123		
Province									
Kinshasa	154	42.2	0.418	215	59.1	0.294	67	18.5	0.008
Kasai Oriental	91	45.7		109	54.5		56	28.3	
Sociodemographic characteristics									
Sex									
Male	91	39.9	0.164	118	51.5	0.019	31	13.7	0.000
Fémale	154	45.8		206	61.5		92	27.5	
Age group									
10–19 years old	105	43.0	0.865	133	54.1	0.153	57	23.5	0.455
20–24 years old	140	43.8		191	60.1		66	20.8	
Marital status									
Single	212	43.2	0.671	282	57.3	0.946	104	21.3	0.300
Married, Widowed, Divorced, Separated	33	45.8		41	57.8		19	26.8	
Level of education									
No education	16	32.7	0.344	30	62.5	0.363	10	20.8	0.374
Primary	14	37.8		23	62.2		10	27.8	
Secondary	165	45.3		199	54.5		84	23.3	
Higher education	47	43.1		68	62.4		18	16.4	
Cohabitation with biological parents (father and mother)									
Both parents	108	38.3	0.026	155	54.6	0.338	56	19.8	0.389
Father or Mother	56	52.8		62	58.5		27	26.0	
None	81	46.0		107	61.5		40	23.1	
Participation in religious services (worship/mass/prayer)									
More than once a week	166	43.9	0.745	212	56.1	0.351	87	23.3	0.272
Other	79	42.5		112	60.2		36	19.3	
Access to the internet									
Often/Rarely	159	44.4	0.539	208	57.9	0.755	75	21.0	0.469
Never used the internet, Unfamiliar with the internet	86	41.8		116	56.6		48	23.7	
Membership in a social group (association, club)									
Yes	80	45.5	0.532	105	60.0	0.429	43	24.6	0.323
No	165	42.6		219	56.4		80	20.8	
Communication about sexuality or intimate subjects									
Communication about sexuality or intimate subjects with a family member									
Yes	123	42.1	0.548	178	60.8	0.091	63	21.7	0.994
No	117	44.7		140	53.6		56	21.6	
Communication about sexuality or intimate subjects with someone outside the family									
Yes	190	45.1	0.086	249	59.1	0.061	97	23.3	0.298
No	44	36.4		60	49.6		23	18.9	
Adolescents’ perceptions									
I believe we can trust people in my community									
Agree	146	41.2	0.172	202	57.1	0.810	76	21.7	0.854
Disagree	99	47.1		122	58.1		47	22.4	
									
I feel safe in my community									
Agree	179	43.6	0.978	236	57.3	0.943	91	22.2	0.856
Disagree	66	43.4		87	57.6		32	21.5	
It's the man who decides when to have sexual relations									
Agree	170	42.3	0.311	233	58.1	0.448	87	21.8	0.719
Disagree	72	47.1		84	54.6		35	23.2	
A man needs to have multiple sexual partners, even if he has a regular sexual partner									
Agree	152	49.5	0.005	188	61.0	0.052	82	26.9	0.004
Disagree	81	37.2		114	52.5		35	16.2	
A woman should tolerate violence from her sexual partner or spouse to avoid losing him									
Agree	91	56.2	0.001	101	61.6	0.242	47	29.6	0.011
Disagree	138	39.9		193	56.1		67	19.4	

Considering unwanted touching, adolescents cohabitating with only one parent (father or mother) are proportionally more numerous (52.8%) to have experienced it. They are followed by adolescents who do not reside with any parent (46%) and those who live in the same household as both parents (38.3%). Adolescents who have communicated about sexuality or intimate subjects with someone outside the family are more likely to experience unwanted touching (45.1%) compared to those who have not communicated (36.4%). Regarding adolescents' perceptions about the role and place of partners in intimate relationships, those who agree with certain statements are more likely to experience unwanted touching than those who do not agree: “A man needs to have multiple sexual partners, even if he has a regular sexual partner” (49.5% vs. 37.2%) and “A woman should tolerate violence from her sexual partner or spouse in order not to lose him” (56.2% vs. 39.9%).

Regarding attempted forced sexual intercourse, girls are more likely (61.5%) to have experienced such an attempt than boys (51.5%). Adolescents who have communicated about sexuality or intimate subjects with someone outside the family are more likely to experience attempted forced sexual intercourse (60.8%) compared to those who have not communicated (53.6%). The same applies to communication about subjects with someone outside the family (59.1% vs. 49.6%). Adolescents who agree with a certain statement are more likely to experience attempted forced sexual intercourse than those who do not agree (61% vs. 52.5%): “A man needs to have multiple sexual partners, even if he has a regular sexual partner”.

The results show that adolescents from Kasai Oriental are more likely (28.3%) to have been physically forced, injured, or threatened to have sexual intercourse than those from Kinshasa (18.5%). Girls are more likely (27.5%) to have been physically forced, injured, or threatened to have sexual intercourse than boys (13.7%). Regarding adolescents’ perceptions, those who agree with certain statements are more likely to have been physically forced, injured, or threatened to have sexual intercourse than those who do not agree. These statements include “A man needs to have multiple sexual partners, even if he has a regular sexual partner” (26.9% vs. 16.2%) and “A woman should tolerate violence from her sexual partner or spouse in order not to lose him” (29.6% vs. 19.4%).

### Factors associated with non-consensual sexual acts among adolescents

Table 4 presents the results of binomial logistic regression models predicting the probability of experiencing non-consensual sexual acts among sexually active adolescents. The independent variables are related to sociodemographic characteristics, communication about sexuality or intimate subjects, and adolescents' perceptions. Only factors associated with the 10% threshold are interpreted. Factors associated with unwanted touching include gender, cohabitation with biological parents (father and mother), age at first sexual intercourse, and the statements “A man needs to have multiple sexual partners, even if he has a regular sexual partner” and “A woman should tolerate violence from her sexual partner or spouse in order not to lose him.” For attempted forced sexual intercourse, associated factors include gender, age at first sexual intercourse, and communication about sexuality or intimate subjects with a family member. Regarding forced sexual intercourse, associated factors include province, gender, cohabitation with biological parents (father and mother), and the statements “It is the man who decides when to have sexual relations,” “A man needs to have multiple sexual partners, even if he has a regular sexual partner,” and “A woman should tolerate violence from her sexual partner or spouse in order not to lose him.”

**Table 4:** Logistic regression coefficients predicting the probability of experiencing non-consensual sexual acts among adolescents in the provinces of Kinshasa and Kasai oriental (Democratic Republic of the Congo), 2018.

Variables related to sociodemographic characteristics, communication about sexuality or intimate subjects, and adolescents’ perceptions		Has experienced non-consensual touching		Has experienced an attempted forced sexual intercourse		Has experienced physical force, injury, or threats for sexual intercourse	
		Coefficient	Standard errors	Coefficient	Standard errors	Coefficient	Standard errors
Province	Kinshasa	0.000		0.000		0,000	
	Kasai Oriental	0.383	(0.247)	−0.109	(0.241)	0.683**	(0.291)
Sociodemographic characteristics							
Sex	Male	0.000		0.000		0.000	
	Female	0.605***	(0.225)	0.594***	(0.217)	1.115***	(0.282)
Age group	10–19 years old	0.000		0,000		0.000	
	20–24 years old	0.00863	(0.231)	0.243	(0.227)	−0.110	(0.269)
Marital status	Single	0.000		0.000		0.000	
	Married/Widowed/Divorced/Separated	−0.385	(0.347)	−0.251	(0.344)	−0.200	(0.382)
Level of education	No education	0.000		0,000		0.000	
	Primary	0.151	(0.548)	−0.377	(0.526)	0.0188	(0.593)
	Secondary	0.561	(0.399)	−0.300	(0.384)	−0.00312	(0.446)
	Higher education	0.555	(0.450)	0.0296	(0.436)	−0.304	(0.529)
Cohabitation with biological parents (father and mother)	Both parents	0.000		0.000		0.000	
	Father or Mother	0.887***	(0.277)	0.345	(0.272)	0.539*	(0.324)
	None	0.654***	(0.248)	0.198	(0.242)	0.383	(0.292)
Participation in religious services (worship/mass/prayer)	More than once a week	0.000		0.000		0.000	
	Other	0.195	(0.215)	0.325	(0.211)	−0.109	(0.258)
Access to the internet	Often/Rarely	0.000		0.000		0.000	
	Never used the internet/Unfamiliar with the internet	−0.191	(0.41)	0.0859	(0.237)	0.00589	(0.280)
Membership in a social group (association, club)	Yes	0.000		0.000		0.000	
	No	−0.0462	(0.219)	−0.174	(0.217)	−0.345	(0.252)
Communication about sexuality or intimate subjects							
Age at the first intercourse		−0.116**	(0.0483)	−0.129***	(0.0476)	−0.0458	(0.0580)
Communication about sexuality or intimate subjects with a family member	Yes	0.000		0.000		0.000	
	No	−0.0740	(0.204)	−0.375*	(0.199)	0.0821	(0.243)
Communication about sexuality or intimate subjects with someone outside the family	Yes	0.000		0.000		0.000	
	No	−0.292	(0.258)	−0.240	(0.251)	−0.446	(0.308)
Adolescents' perceptions							
I believe we can trust people in my community	Agree	0.000		0.000		0.000	
	Disagree	0.379	(0.239)	−0.0569	(0.234)	0.0652	(0.286)
I feel safe in my community	Agree	0.000		0.000		0.000	
	Disagree	−0.0635	(0.254)	−0.155	(0.248)	0.0437	(0.304)
It's the man who decides when to have sexual relations	Agree	0.000		0.000		0.000	
	Disagree	0.367	(0.235)	−0.186	(0.230)	0.465*	(0.283)
A man needs to have multiple sexual partners, even if he has a regular sexual partner	Agree	0.000		0,000		0.000	
	Disagree	−0.428**	(0.214)	−0.224	(0.209)	−0.596**	(0.261)
A woman should tolerate violence from her sexual partner or spouse to avoid losing him	Agree	0.000		0.000		0.000	
	Disagree	−0.890***	(0.242)	−0.255	(0.238)	−0.577**	(0.283)
Constant		1.076	(0.892)	2.701***	(0.893)	−0.790	(1.068)
Observations		467		468		465	

* *p* < 0.1.

** *p* < 0.05.

*** *p* < 0.01.

**Table 5:** Knowledge of an assistance source for victim of forced sexual intercourse in the provinces of Kinshasa and Kasai oriental (Democratic Republic of the Congo), 2018.

	Knowledge of an institution/person who can provide assistance to a person victim of forced sexual intercourse			
	Yes		No	
	*n*	%	*n*	%
Sex				
Male	8	25,8	23	74.2
Female	37	40.7	54	59.3
Province				
Kinshasa	29	43.3	38	56.7
Kasai Oriental	16	29.1	39	70.9
**Total**	**45**	**36**.**9**	**77**	**63**.**1**

### Utilization of appropriate services for the care of victims of sexual violence

Among adolescents who have been physically forced, injured, or threatened to have sexual intercourse, 36.9% reported knowing an institution or person who can assist a victim of forced sexual intercourse (Table 5). Girls are proportionally more numerous (40.7%) in knowing the institution or person than boys (25.8%). The same applies to adolescents from Kinshasa (43.3%) compared to those from Kasai Oriental (29.1%). Of the 12 adolescents aware of a source of assistance for victims of forced sex, a quarter (25.5%) of adolescents who have been victims of forced sexual intercourse and are aware of an appropriate institution or person have sought help from a professional for the violence experienced. Among those who sought help, the majority (90.9%) received it from a professional.

**Figure 1:**
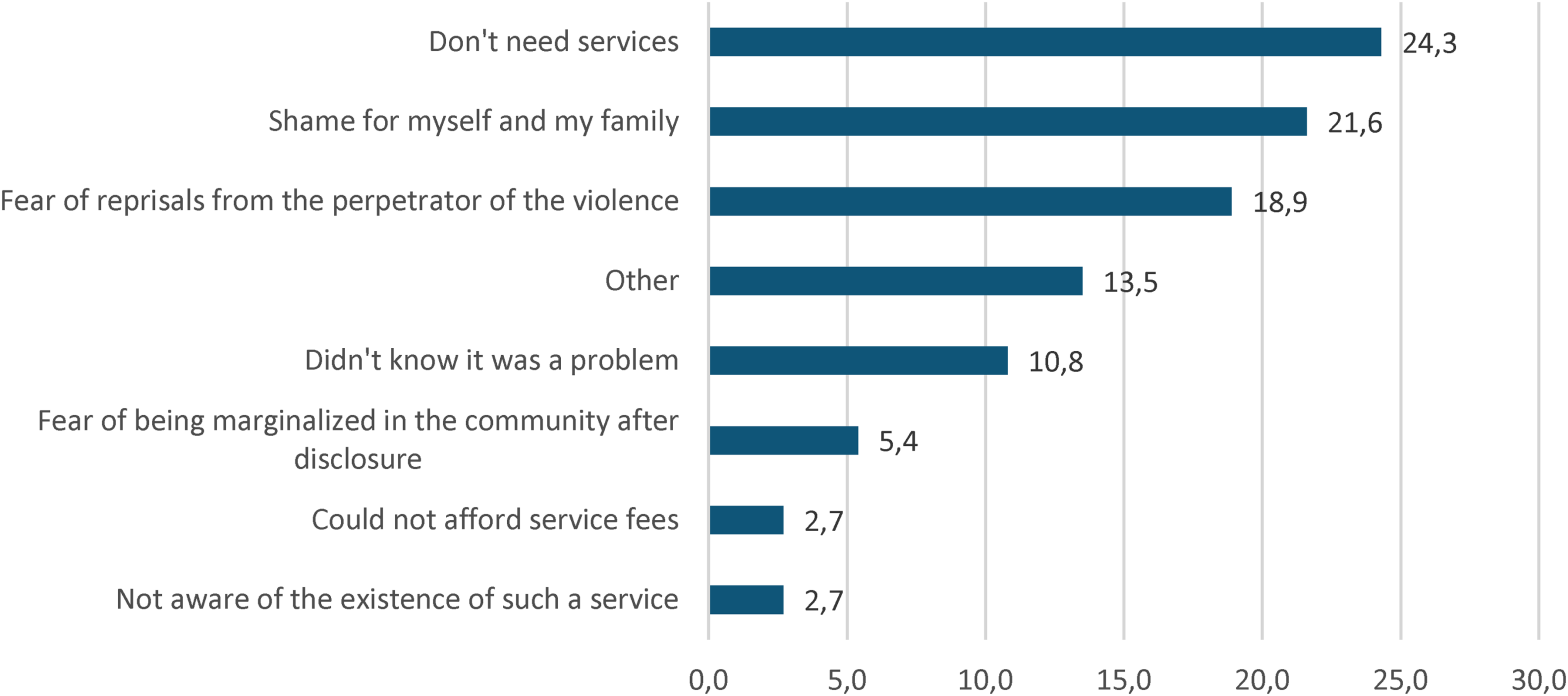
Reasons for not seeking assistance from personnel in a service.

When asked about the reasons for not seeking help from personnel in an appropriate service (Figure 1), the concerned adolescents cited several reasons, with the main ones being no need for such a service (24.3%), shame for oneself, and one's family (21.6%), and fear of retaliation from the perpetrator of the violence (18.9%). One-tenth (10.8%) stated not knowing that experiencing sexual violence is a problem.

## Discussion

The median age of sexual debut among adolescents aged 10–24 participating in the baseline survey of the “Reducing the vulnerability of adolescents and young girls to violence and HIV/AIDS infection” project is 15 years. This result reflects an earlier sexual initiation among a significant portion of these adolescents, with potentially negative implications for their future behavior (19, 20). However, the description of the characteristics of adolescents who have initiated sexual activity reveals that the majority are educated (at least to a secondary level), attend religious services, have access to the Internet, and are confident in their community. At least half have communicated about sexuality or intimate subjects. These are adolescents who are more likely to be informed about sexuality and its consequences and who, for the most part, reject violence against women in intimate relationships, even though many believe in the dominant role of men in an intimate relationship.

Overall, among adolescents aged 10–24 in the survey population, the prevalence of non-consensual sexual acts ranges from 5.8% to 15.3%, depending on the type of act. Forced sexual intercourse is the least prevalent, while attempted forced sexual intercourse remains the most prevalent. These prevalences are similar to those observed in a study conducted by the Ministry of Women, Family, and Children in Côte d'Ivoire (14), which explored the same types of non-consensual sexual acts in the general population aged 18–24. Among adolescents aged 10–24 in the provinces of Kinshasa and Kasai Oriental who have initiated sexual activity, the prevalence of non-consensual sexual acts is higher, ranging from 22.0% to 57.4%, corresponding, respectively, to the experience of forced sexual intercourse and attempted forced sexual intercourse. Considering the proportion of adolescents who reported having experienced forced sexual intercourse, this is higher than the proportions obtained in previous studies conducted in Africa. This corroborates with findings from Rwanda among upper secondary school students, which is 15.5% (6), from Benin City, Nigeria, among university students, which is 18.4% (7), and from Nyeri, Kenya, among young women (21%) and men (11%) (8).

The factors associated with non-consensual sexual acts are related to sociodemographic characteristics, communication about sexuality or intimate subjects, and adolescents' perceptions of the role and place of partners in intimate relationships. Regarding sociodemographic characteristics, these include gender, cohabitation with biological parents (father and mother), and age at first sexual intercourse. Communication about sexuality or intimate subjects with a family member and all variables related to adolescents' perceptions of the role and place of partners in intimate relationships are associated with non-consensual sexual acts.

The tendency to attach great importance to traditional values leads to the acceptance of sexual coercion by adolescent victims (6). The results of this study indicate a low interest among victims in seeking professional help for the violence they have experienced. Only 25.5% of adolescents who were aware of an institution or individual who could assist actually sought it. As observed in studies conducted in Côte d'Ivoire and Burkina Faso, there is a trend towards not reporting perpetrators and/or not seeking appropriate care services (14, 15).

The results of the study confirm the existence of non-consensual sexual acts among adolescents in the DRC. Because of the immediate and future consequences of sexual violence, it is necessary to combat this phenomenon from adolescence onwards. Unfortunately, the DRC is not yet sufficiently committed to this approach. Admittedly, the phenomenon is well targeted in the National Adolescent Health Policy (PNSA) and in Law 06/018 of July 20, 2006, but it is poorly taken into account in the operational sphere of the health system. And yet, it is this first level that must do most of the work in preventing the phenomenon and caring for victims of sexual violence. There is also a void in the educational and social sectors. The few projects working in the field of sexual violence tend to target armed conflict zones, and ignore adolescents.

One of the strategies for dealing with non-consensual sexual acts among adolescents is to identify and work with the whole of the adolescent's living environment, both in and out of school. This includes the household, the school, the health center, the socio-educational center, the police, the justice system and the community. This means involving all the players in the adolescents' living environment, while offering services adapted to these contexts and to the adolescents' needs. The first step is to train the adults working in these environments, to raise awareness of the scale of the phenomenon and the means of preventing and combating it. We need to deconstruct the social and cultural logics behind non-consensual sexual acts, and build sexual and reproductive health education that respects each partner. Next, we need to raise awareness and train adolescents, and set up self-managed structures to give them a sense of responsibility in preventing and combating non-consensual sexual acts. Once all players, including adolescents, have been informed and trained, a regular system of prevention, denunciation and combating is put in place, followed by referral and care for victims. This is the strategy put in place by the pilot project to reduce the vulnerability of adolescent girls and young women in the DRC, and it is likely to be a long-term one because of its roots among adolescents and in the community.

## Conclusion

The prevalence of non-consensual sexual acts among the general adolescent population (aged 10–24) or among those who have initiated sexual activity is higher in the cities of Kinshasa and Mbuji-Mayi in the DRC. This situation is observed among educated adolescents, who are likely to be better informed about sexuality and its consequences but may also be more attached to traditional beliefs about the dominant role of men in intimate relationships. Additionally, various barriers prevent victims of sexual violence from seeking appropriate care. The study results advocate for mobilizing community and family spheres in the fight against sexual violence and providing health services tailored to the needs of adolescents and adapted to socio-cultural contexts.

## Data Availability

The raw data supporting the conclusions of this article will be made available by the authors, without undue reservation.
